# Better cancer specific survival in young small cell lung cancer patients especially with AJCC stage III

**DOI:** 10.18632/oncotarget.16823

**Published:** 2017-04-04

**Authors:** Haiyong Wang, Jingze Zhang, Fang Shi, Chenyue Zhang, Qinghua Jiao, Hui Zhu

**Affiliations:** ^1^ Department of Radiation Oncology, Shandong Cancer Hospital and Institute, Shandong Cancer Hospital Affiliated to Shandong University, Shandong Academy of Medical Sciences, Jinan 250117, China; ^2^ Department of Integrative Oncology, Fudan University Shanghai Cancer Center, Shanghai 200032, China; ^3^ Cancer Center, The Second Hospital of Shandong University, Shandong 250033, China

**Keywords:** small cell lung cancer, young, cancer specific survival, SEER

## Abstract

It has been reported that younger patients with non-small cell lung cancer (NSCLC) tend to have a better prognosis. Yet, few studies have focused on the clinicopathological characteristics and prognosis of young small cell lung cancer (SCLC), especially for patients with age < 50. In our study, we used Surveillance, Epidemiology, and End Results (SEER) population-based data and identified 16503 patients with SCLC including 711 patients aged < 50, 3338 patients aged 50–59, 5937 patients aged 60–69, 4649 patients aged 70–79 and 1868 patients aged ≥ 80 between 2010 and 2013. The Kaplan-Meier methods was used to develop the survival curve, and the results showed that the SCLC patients with aged < 50 tended to a better over survival (OS) and cancer specific survival (CSS) (all, *P* < 0.001). In addition, Cox regression model was used to analyze survival prognosis factors and perform subgroup analysis. The results showed that age was an independent prognostic factor for CSS (*P* < 0.001). Importantly, we found that for the patients with AJCC stage III subgroup, the age < 50 had apparent CSS benefit compared with any other age group (all, *P* < 0.01). Interestingly, for the patients with no surgery, radiation and no radiation subgroup, the age < 50 had no apparent CSS benefit only compared with age 50–59 (all, *P* > 0.05). In conclusion, our study demonstrated that the SCLC patients with aged < 50 tended had a better survival benefit, especially for patients with AJCC stage III.

## INTRODUCTION

As one of the most common malignancies, lung cancer stands out as the main cause of cancer mortality worldwide [1]. Small cell lung cancer (SCLC), representing approximately 15% of clinical lung cancer cases [2], is an aggressive subtype characterized by rapid proliferation and early metastasis [3]. Despite a relatively high response rate upon initial treatment, 5-year survival rate remains at a 6% low level due to its inclination to relapse [4].

Disease extent is one of the most established prognostic factors. The median survival for patients with limited-stage disease (LD) ranges from 15 to 20 months, almost twice as that for patients with extensive-stage disease (ED) [5]. At present, many factors such as sex, performance status, and several routine laboratory tests have been demonstrated to have certain influence on prognosis [6–10]. However, no histological or molecular features have been identified to be of prognostic use so far [11]. It is worth noting that age has been recognized as a significant prognostic factor in some tumors. In non-small cell lung cancer, younger patients take advantage in overall and relative survival, particularly at earlier stage [12]. By contrast women with breast cancer aged 35 years or younger are recommended to adopt additional risk-reduction strategies after radical treatment [13]. However, there are no established conclusions about the prognostic significance of age in SCLC so far. Previous studies lack direct comparison of outcomes between the young and the old because of the dominant proportion of elderly population in patients with SCLC [14, 15]. To ascertain this issue, we used the Surveillance, Epidemiology, and End Results (SEER) registries, which covered an adequate number of patients of all ages, to determine the prognostic value of age on patients with SCLC.

## RESULTS

### Patient characteristics

A total of 16503 patients with SCLC were identified in SEER database during the period from 2010 to 2013. The white race accounted for a large majority (84.4% for age < 50, 85.1% for age 50–59, 86.5% for age 60–69, 87.2% for age 70–79, 87.2% for age ≥ 80) and the proportion of men and women was roughly equal (all, 50% or so). More than three quarters of patients had unclear grading (all, 76% or so). More patient were diagnosed in AJCC stage IV (68.8% for age < 50, 70.1% for age 50–59, 70.8% for age 60–69, 71.1% for age 70–79, 72.9% for age ≥ 80). Few patients underwent surgery (2.4% for age < 50, 2.4% for age 50–59, 2.0% for age 60–69, 1.9% for age 70–79, 1.1% for age ≥ 80). In addition, most patients with age < 50 received radiation (64.1% VS. 35.9%), and most patients with age ≥ 80 did not receive radiation (24.6% VS. 75.4%) The detailed statistical results were shown in Table 1.

**Table 1:** Characteristics of Patients from SEER Database according to different age group

Characteristics	< 50	50–59	60–69	70–79	≥ 80	*P*
**Total**	711	3338	5937	4649	1868	
**Race**						< 0.001
White	600 (84.4)	2842 (85.1)	5138 (86.5)	4055 (87.2)	1628 (87.2)	
Black	88 (12.4)	389 (11.7)	550 (9.3)	359 (7.7)	129 (6.9)	
Others	23 (3.2)	107 (3.2)	249 (4.2)	235 (5.1)	111 (5.9)	
**Sex**						0.046
Male	339 (47.7)	1694 (50.7)	3056 (51.5)	2301 (49.5)	902 (48.3)	
Female	372 (52.3)	1644 (49.3)	2881 (48.5)	2348 (50.5)	966 (51.7)	
**Grade**						0.060
I–II	7 (1.0)	4 (0.1)	20 (0.3)	10 (0.2)	5 (0.3)	
III	62 (8.7)	267 (8.0)	514 (8.7)	401 (8.6)	148 (7.9)	
IV	103 (14.5)	481 (14.4)	851 (14.3)	695 (14.9)	273 (14.6)	
Unknown	539 (75.8)	2586 (77.5)	4552 (76.7)	3543 (76.2)	1442 (77.2)	
**AJCC stage**						< 0.001
I–II	25 (3.5)	154 (4.6)	313 (5.3)	301 (6.5)	117 (6.3)	
III	197 (27.7)	844 (25.3)	1420 (23.9)	1044 (22.5)	389 (20.8)	
IV	489 (68.8)	2340 (70.1)	4204 (70.8)	3304 (71.1)	1362 (72.9)	
**Radiation**						< 0.001
Yes	456 (64.1)	1893(56.7)	2949 (49.7)	1895 (40.8)	460 (24.6)	
No	255 (35.9)	1445 (43.3)	2988 (50.3)	2754 (59.2)	1408 (75.4)	
**Surgery**						0.023
Yes	17(2.4)	79 (2.4)	118 (2.0)	89 (1.9)	20 (1.1)	
No	694 (97.6)	3259 (97.6)	5819 (98.0)	4560 (98.0)	1848 (98.9)	

### Impact of age on survival outcomes

**Figure 1:**
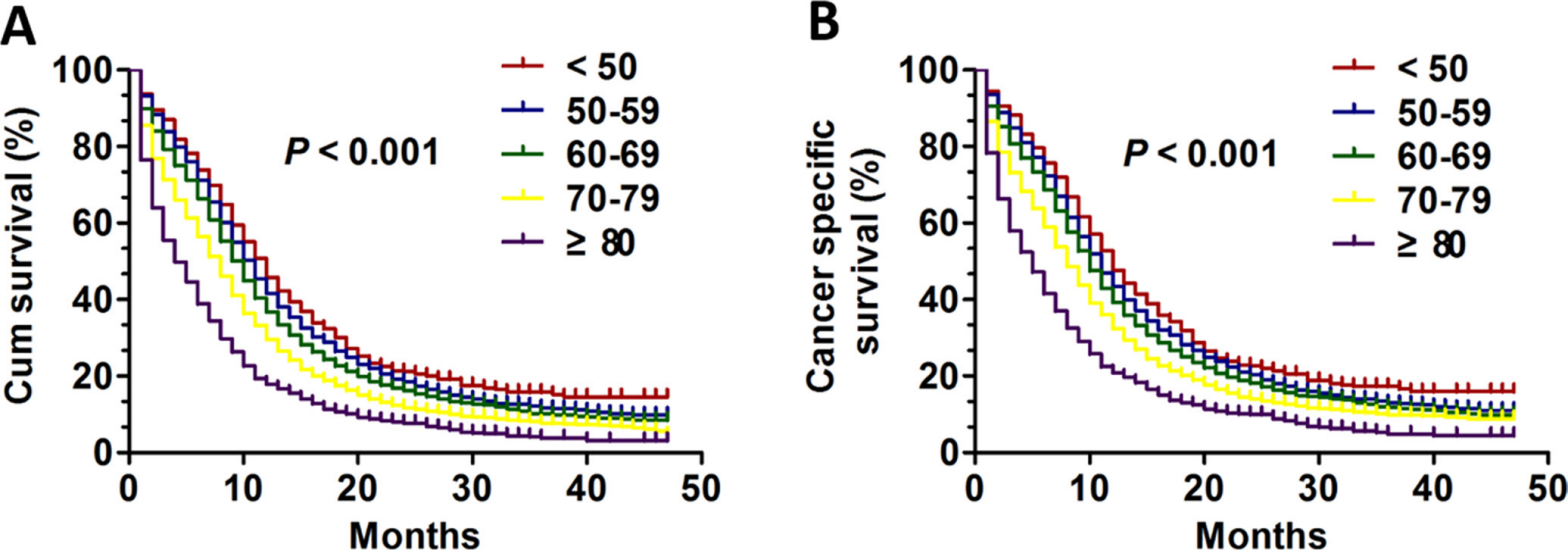
The survival curves in SCLC patients of different age groups between 2010 and 2013 (**A**) The OS curves: (*P* < 0.001); (**B**) The CSS curves (*P* < 0.001).

To compare the influence of age on the prognosis of patients with SCLC, patients were divided into five different groups including age < 50, 50–59, 60–69, 70–79 and ≥ 80. The prognoses of different groups was further analyzed using Kaplan-Meier estimates. As showed in Figure 1, the young group aged < 50 tended had best outcomes with improved OS (*P* < 0.001) and CSS (*P* < 0.001). In addition, we found that the clinicopathological differences among the five age groups might have result in the differences of the prognosis. Next, we regarded CSS as our primary study end point. The univariate analysis and multivariate Cox regression model was further applied and showed that age still was an independent prognostic factor for CSS (*P* < 0.001). Importantly, other several variables were also validated as independent prognostic factors for CSS in these patients. These prognostic factors included race (*P* = 0.002), sex (*P* < 0.001), AJCC stage (*P* < 0.001), surgery (*P* < 0.001) and radiation (*P* < 0.001). The detailed statistical results were showed in Table 2.

**Table 2:** Univariate and multivariate Cox proportional hazards regression models to evaluate the prognostic factors for CSS from SEER Database

	Univariate analyses		Multivariate analyses		
Variable	Wald χ^2^	*P*	HR	95% CI	*P*
**Age**	658.7	< 0.001			< 0.001
< 50			Reference		
50–59			1.068	0.967–1.180	0.197
60–69			1.195	1.086–1.315	< 0.001
70–79			1.459	1.323–1.608	< 0.001
≥ 80			1.992	1.793–2.213	< 0.001
**Race**	14.9	0.001			0.002
White			Reference		
Black			0.922	0.864–0.983	0.013
Others			0.883	0.804–0.968	0.008
**Sex**	60.7	< 0.001			< 0.001
Male			Reference		
Female			0.902	0.870–0.935	< 0.001
**Grade**	30.7	< 0.001			0.304
I			Reference		
II			0.954	0.455–1.998	0.900
III			1.341	0.759–2.371	0.312
IV			1.367	0.775–2.413	0.280
Unknown			1.389	0.788–2.448	0.255
**AJCC stage**	1660.0	< 0.001			< 0.001
I			Reference		
II			1.511	1.225–1.865	< 0.001
III			2.240	1.895–2.647	< 0.001
IV			4.224	3.584–4.977	< 0.001
**Surgery**	139.9	< 0.001			< 0.001
Yes			Reference		
No			1.764	1.486–2.094	< 0.001
**Radiation**	1906.1	< 0.001			< 0.001
Yes			Reference		
No			1.877	1.804–1.952	< 0.001

### Subgroup analysis of age on CSS based on different stages

**Figure 2:**
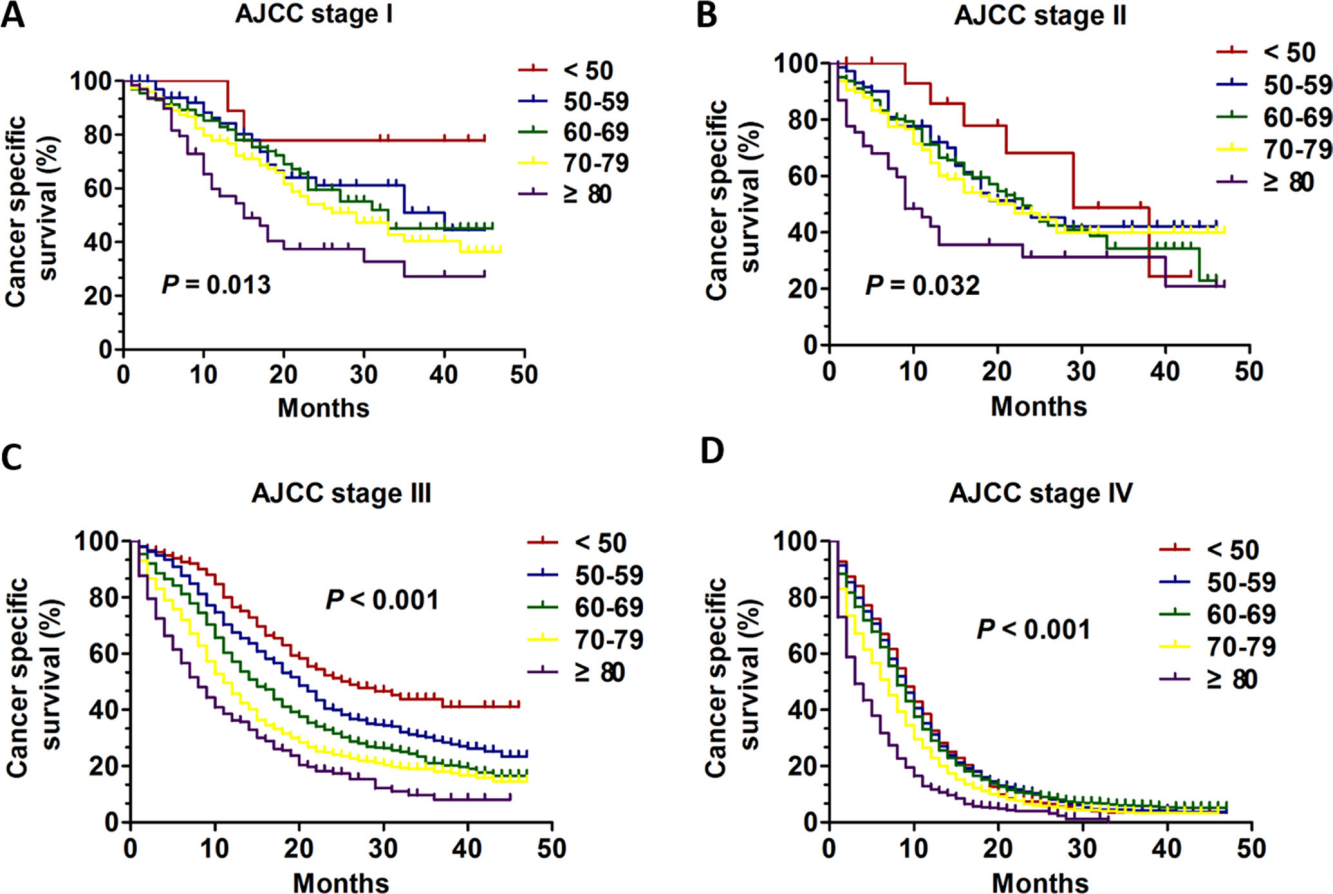
The survival curves in SCLC patients of different age groups at each stage between 2010 and 2013 (**A**) Kaplan-Meier curves for CSS of different age groups in stage I: (*P* = 0.013); (**B**) Kaplan-Meier curves for CSS of different age groups in stage II: (*P* = 0.032); (**C**) Kaplan-Meier curves for CSS of different age groups in stage III: (*P* < 0.001); (**D**) Kaplan-Meier curves for CSS of different age groups in stage IV: (*P* < 0.001).

Next, the Kaplan-Meier method and log-rank test were used to excavate the important prognostic factors for CSS according to different age groups. We performed analysis of age on CSS in every AJCC stage. In patients with AJCC stage I and II (Figure 2A, 2B), although the young group aged < 50 tended to had better prognostic with improved CSS (*P* = 0.013 for stage I; *P* = 0.032 for stage II), there was no obvious clinical significance due to the small sample size in young patients and inconspicuous *P* value. Interestingly, in patients with AJCC stage III and stage IV (Figure 2C and 2D), the overall CSS statistics difference was found (all, *P* < 0.001). Importantly, the univariate and multivariate Cox regression model was performed to analysis the prognostic value of age in specific stages. Only in patients with AJCC stage III and IV, age was validated as an independent prognostic factor (Table 3). For the patients with AJCC stage III, the patients aged < 50 had apparent CSS benefit compared with any other age group (age 50–59 VS age < 50: HR:1.418; 95% CI: 1.104–1.798, *P* = 0.006; age 60–69 VS age < 50: HR:1.772; 95% CI: 1.401–2.241, *P* < 0.001; age 70–79 VS age < 50: HR:2.128; 95% CI: 1.677–2.699, *P* < 0.001; age ≥ 80 VS age < 50: HR: 2.616; 95% CI: 2.024–3.382, *P* < 0.001) (Table 3). However, for the patients with AJCC stage IV, the patients aged < 50 had apparent CSS benefit only compared with two age group (age 70–79 VS age < 50: HR:1.324; 95% CI: 1.189–1.475, *P* < 0.001; age ≥ 80 VS age < 50: HR: 1.830; 95% CI: 1.628–2.057, *P* < 0.001) (Table 3). Additionally, we compared CSS of different age groups in stage III and stage IV. In stage III the 1-year CSS of different group was 76.5%, 67.5%, 57.2%, 45.7% and 36.2% respectively and 3-years CSS of five groups was 43.8%, 29.1%, 21.0%, 18.0% and 8.0% respectively (Figure 5). In stage IV the 1-year CSS of different group was 32.6%, 31.3%, 29.0%, 22.9% and 11.7% respectively and 2-years CSS of five groups was 7.3%, 10.3%, 9.8%, 6.0% and 4.0% respectively (Figure 5).

**Figure 3:**
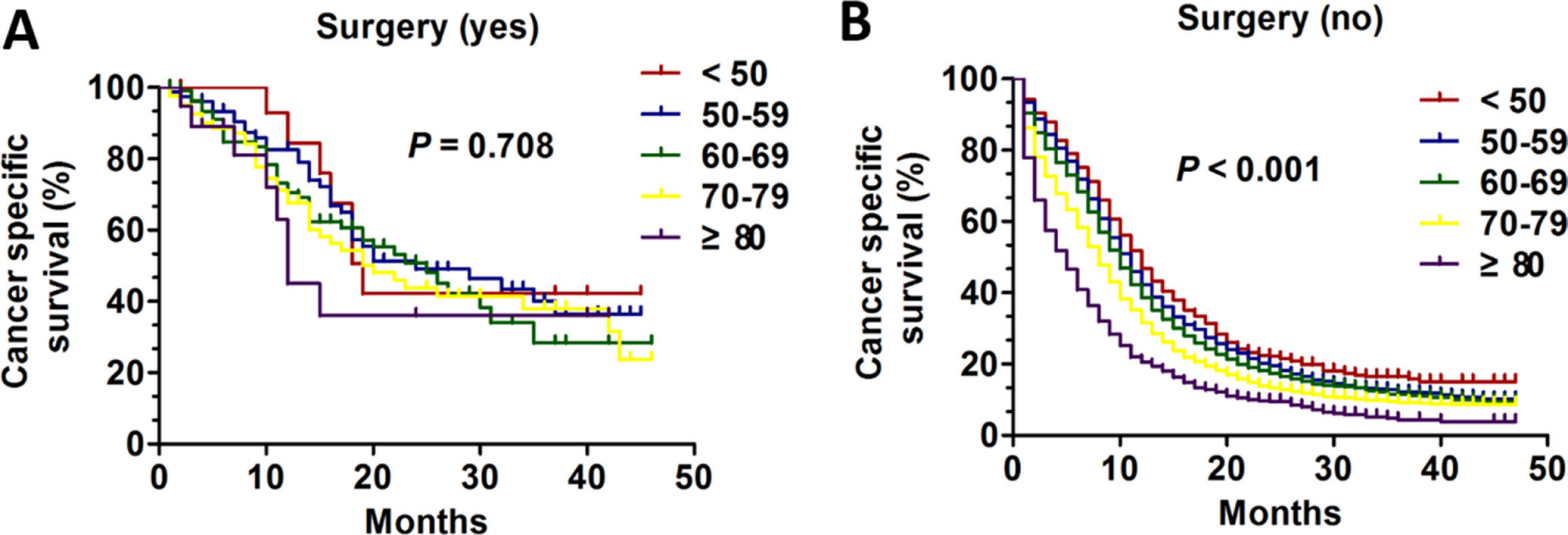
The survival curves in SCLC patients of different age groups according to surgery history between 2010 and 2013 (**A**) Kaplan-Meier curves for CSS of different age groups with surgical treatment (*P* = 0.708): (**B**) Kaplan-Meier curves for CSS of different age groups with non-surgical treatment (*P* < 0.001).

**Table 3:** Univariate and multivariate Cox proportional hazards regression models to evaluate the effect of age for CSS according to different stages

	Univariate analyses		Multivariate analyses		
Variable	Wald χ^2^	*P*	HR	95% CI	*P*
**AJCC stage I**					
**Age**	15.2	0.004			0.058
< 50			Reference		
50–59			2.437	0.482–12.320	0.281
60–69			2.198	0.448–10.779	0.332
70–79			2.789	0.564–13.787	0.208
≥ 80			4.242	0.841–21.391	0.080
**AJCC stage II**					
Age	8.9	0.064			Not included
< 50					
50–59					
60–69					
70–79					
≥ 80					
**AJCC stage III**					
Age	232.5	< 0.001			< 0.001
< 50			Reference		
50–59			1.408	1.104–1.798	0.006
60–69			1.772	1.401–2.241	< 0.001
70–79			2.128	1.677–2.699	< 0.001
≥ 80			2.616	2.024–3.382	< 0.001
**AJCC stage IV**					
Age	519.9	< 0.001			< 0.001
< 50			Reference		
50–59			0.995	0.890–1.111	0.923
60–69			1.082	0.973–1.203	0.147
70–79			1.324	1.189–1.475	< 0.001
≥ 80			1.830	1.628–2.057	< 0.001

**Figure 4:**
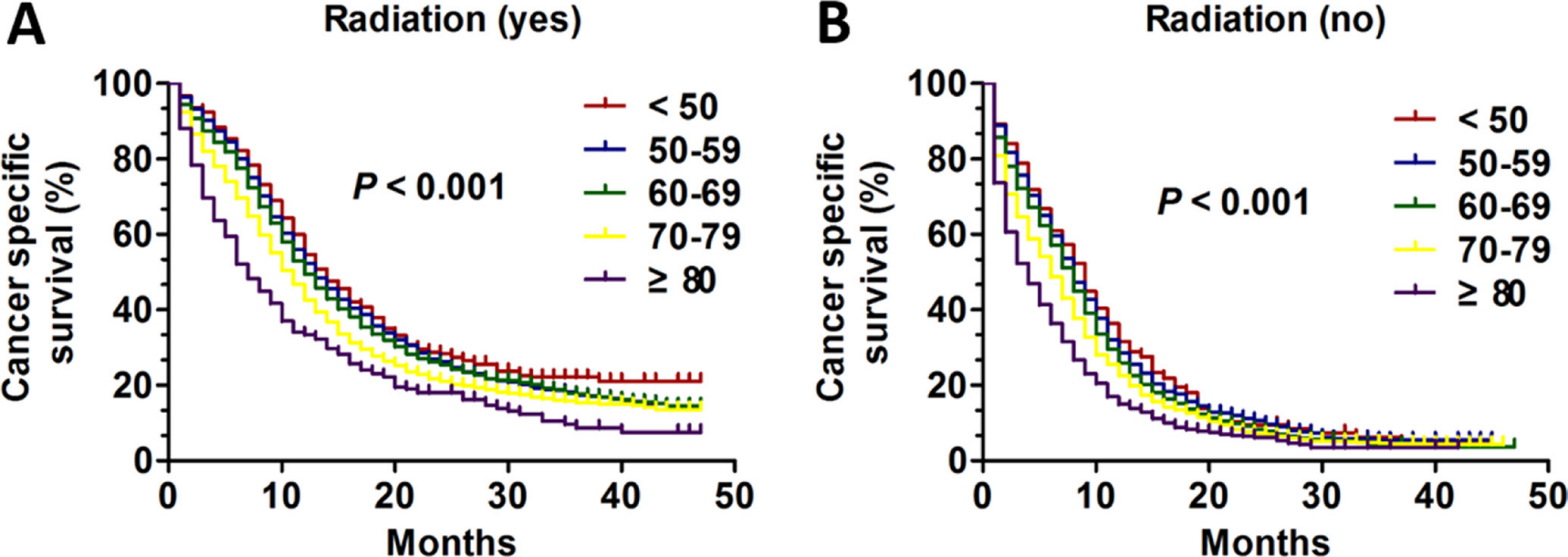
The survival curves in SCLC patients of different age groups according to radiation history between 2010 and 2013 (**A**) Kaplan-Meier curves for CSS of different age groups with radiation treatment (*P* < 0.001): (**B**) Kaplan-Meier curves for CSS of different age groups with no radiation treatment (*P* < 0.001).

**Figure 5:**
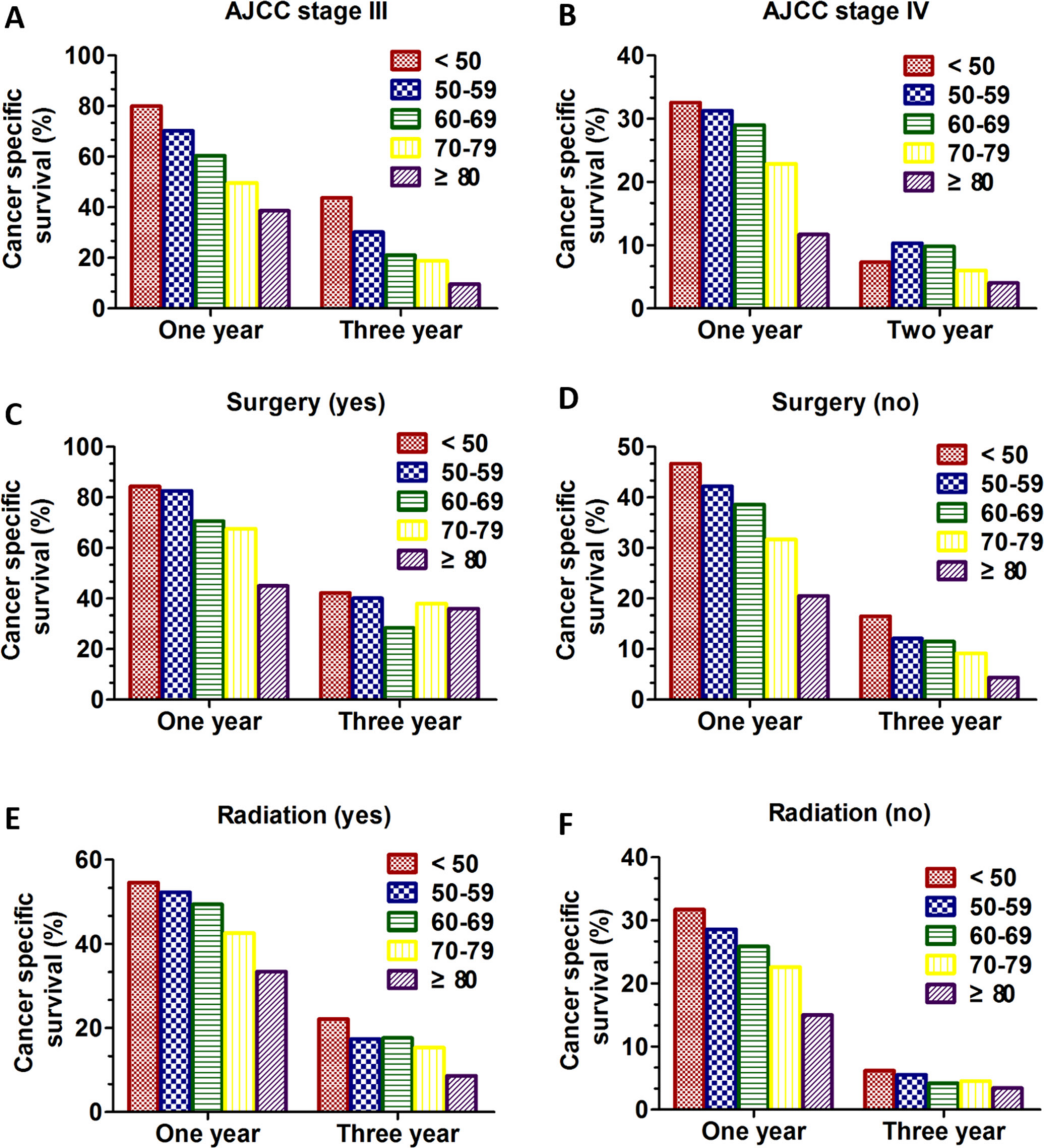
1-year and 3-year CSS in SCLC patients of different age groups in different subgroups between 2010 and 2013 (**A**) 1-year and 3-year CSS in SCLC patients with stage III; (**B**) 1-year and 2-year CSS in SCLC patients with stage IV; (**C**) 1-year and 3-year CSS in SCLC patients who underwent surgical treatment; (**D**) 1-year and 3-year CSS in SCLC patients who underwent non-surgical treatment. (**E**) 1-year and 3-year CSS in SCLC patients who underwent radiation treatment. (**F**) 1-year and 3-year CSS in SCLC patients who underwent no radiation treatment.

### Subgroup analysis of age on CSS based on history of surgery

We also performed analysis of age on CSS according to surgical history using the Kaplan-Meier method and log-rank test. In patients who underwent surgical treatment, there were no appreciable statistics differences among age groups (*P* = 0.708) (Figure 3A). By contrast, in patients managed non-surgically, the overall CSS statistics difference was found (all, *P* < 0.001) (Figure 3B). Then, the univariate and multivariate Cox regression model was performed to analysis the prognostic value of age in surgery subgroup. Only in patients with no surgery subgroup was validated as an independent prognostic factor (Table 4). Interestingly, the patients aged < 50 had apparent CSS benefit only compared with three age group except for age 50–59 (age 50–59 VS age < 50: HR:1.068; 95% CI: 0.966–1.181, *P* = 0.200; age 60–69 VS age < 50: HR:1.194; 95% CI: 1.084–1.315, *P* < 0.001; age 70–79 VS age < 50: HR:1.454; 95% CI: 1.318–1.603, *P* < 0.001; age ≥ 80 VS age < 50: HR: 1.984; 95% CI: 1.785–2.205, *P* < 0.001) (Table 4). Additionally, we compared CSS of different age groups according to surgery history. In patients who received surgery the 1-year CSS of different group was 84.4%, 82.6%,70.6, 67.6, and 45.0% respectively and 3-years CSS of five groups was 42.2%, 40.1%, 28.4%, 38.0%, and 36.0% respectively. In patients who didn't undergo surgery the 1-year CSS of different group was 46.7%, 42.2%, 38.6%, 31.7%, and 20.5% respectively and 3-years CSS of five groups was 16.5%, 12.1%, 11.5%, 9.2% and 4.4% respectively (Figure 5).

**Table 4:** Univariate and multivariate Cox proportional hazards regression models to evaluate the effect of age for CSS according to the history of surgery

	Univariate analyses		Multivariate analyses		
Variable	Wald χ^2^	*P*	HR	95% CI	*P*
**Surgery (yes)**					
Age	3.0	0.558			Not included
< 50					
50–59					
60–69					
70–79					
≥ 80					
**Surgery (no)**					
Age	647.8	< 0.001			< 0.001
< 50			Reference		
50–59			1.068	0.966–1.181	0.200
60–69			1.194	1.084–1.315	< 0.001
70–79			1.454	1.318–1.603	< 0.001
≥ 80			1.984	1.785–2.205	< 0.001

### Subgroup analysis of age on CSS based on history of radiation

We then performed analysis of age on CSS according to radiation history using the Kaplan-Meier method and log-rank test. No matter the patients who received radiation or not, the overall CSS difference was found (all, *P* < 0.001) in Kaplan-Meier curve (Figure 4A and 4B). Then, the univariate and multivariate Cox regression model was also performed to analysis the prognostic value of age in radiation subgroup. The radiation subgroup and no radiation subgroup were all validated as independent prognostic factors (Table 5). Interestingly, the patients aged < 50 had apparent CSS benefit only compared with three age group except for age 50–59 in the two subgroup (radiation: age 50–59 VS age < 50: HR:1.087; 95% CI: 0.952–1.240, *P* = 0.217; age 60–69 VS age < 50: HR:1.176; 95% CI: 1.036–1.336, *P* = 0.012; age 70–79 VS age < 50: HR:1.491; 95% CI: 1.308–1.700, *P* < 0.001; age ≥ 80 VS age < 50: HR: 2.408; 95% CI: 2.049–2.829, *P* < 0.001; no radiation: age 50–59 VS age < 50: HR:1.056; 95% CI: 0.906–1.231, *P* = 0.487; age 60–69 VS age < 50: HR:1.199; 95% CI: 1.035–1.389, *P* = 0.016; age 70–79 VS age < 50: HR:1.412; 95% CI: 1.219–1.637, *P* < 0.001; age ≥ 80 VS age < 50: HR: 1.792; 95% CI: 1.538–2.088, *P* < 0.001) (Table 5). Additionally, we compared CSS of different age groups according to radiation history. In patients who received radiation the 1-year CSS of different group was 54.6%, 52.3%, 49.4%, 42.6%, and 33.4% respectively and 3-years CSS of five groups was 22.1%, 17.4%, 17.6%, 15.3%, and 8.6% respectively. In patients who didn't undergo radiation the 1-year CSS of different group was 31.7%, 28.6%, 25.9%, 22.6%, and 15.0% respectively and 3-years CSS of five groups was 6.2%, 5.5%, 4.2%, 4.5% and 3.4% respectively (Figure 5).

**Table 5:** Univariate and multivariate Cox proportional hazards regression models to evaluate the effect of age for CSS according to the history of radiation

	Univariate analyses		Multivariate analyses		
Variable	Wald χ^2^	*P*	HR	95% CI	*P*
**Radiation (yes)**					
Age	131.3	< 0.001			< 0.001
< 50			Reference		
50–59			1.087	0.952–1.240	0.217
60–69			1.176	1.036–1.336	0.012
70–79			1.491	1.308–1.700	< 0.001
≥ 80			2.408	2.049–2.829	< 0.001
**Radiation (no)**					
Age	180.8	< 0.001			< 0.001
< 50			Reference		
50–59			1.056	0.906–1.231	0.487
60–69			1.199	1.035–1.389	0.016
70–79			1.412	1.219–1.637	< 0.001
≥ 80			1.792	1.538–2.088	< 0.001

## DISCUSSION

Since the median age of SCLC is almost 70-year-old [16], it has been defined as a cutoff to analyze the prognostic value of age [17–23]. All these studies shared the conclusion that elderly patients had comparable response rate and survival to youth even with potentially suboptimal treatments to minimize toxicity. However, by performing comparison among three age cohorts (< 65 years, 65–74 years and ≥ 75 years) instead of between two age groups (< 70 years and ≥ 70years), Joanna et al. found a different result that response rates and overall survival significantly decreased as age advances [24]. Our study adopted five cohorts of different age groups, the result of which shared similarity with the Joanna’s, demonstrating the improved OS and CSS in younger patients.

The following explanation may be possible: it is better to reflect the effect of age on SCLC prognosis with more accurate and precise classification of the patients. When the cut-off age was set up to 70 years, a substantial portion of the “younger” group is actually elderly. According to our data, the proportion under 50 years of patients was only 4.3%. Hence the survival characteristics of much younger patients were covered up easily by dominant proportion of actually older patients in the same age cohort. Comparison among the five groups of varying ages fully exposed differences of OS and CSS, demonstrating the survival advantages of patients younger than 50-year-old. Many articles focused on LD-SCLC when exploring the effect of age on treatment and survival [17, 18, 20–23] while a few researchers conducted analysis throughout all stages[19]. Both kinds of studies failed to present prognosis of every specific stage in different age brackets. Furthermore, the Veterans Administration Lung Study Group (VALSG) staging system [25] would be a little ambiguous considering a retrospective analysis with adequate sample demonstrated significantly worsening survival with advancing TNM stages [26]. Currently the TNM classification system is highly recommended for its accuracy [27]. Therefore we compared CSS among different age groups at every TNM stage from I to IV.

The effect of different ages on CSS between stage I and II might reflect different treatment in early stage SCLC. In the present study, 33.4% of patients with stage I SCLC performed surgery while only 14.3% of patients at stage II underwent resection. It was commonly recognized that young patients benefited more from surgery than the elder because they could tolerate more extensive resection to achieve eradication. However the conclusion should be interpreted with caution due to the relatively small sample at early stages. It has to be noted that only 25 patients with early stage SCLC were included in young group. Therefore, the small sample could not be enough to expose the potential differences. The most obvious difference of CSS benefiting younger patients was presented at stage III. Young group had significant superior CSS compared to other age groups, probably due to their favorable tolerance and compliance allowing high intensive treatment. It was demonstrated that patients with advanced age were less tolerant to receive treatment depicted in published guidelines than their younger counterpart [28]. Although the combination of chemotherapy and thoracic radiotherapy is the standard regimen for patients with LD [29, 30], the chemo-radiotherapy (CRT) would inevitably result in increased toxicity, especially for the older [21, 22, 31]. Considering their infirmity, many of elderly patients with LD-SCLC would have to adopt suboptimal therapy. An analysis of LD-SCLC using the National Cancer Database found that patients were less likely to undergo CRT with advanced ages and 43.7% of patients older than 70 years received chemotherapy solely [18].

By contrast, the CSS difference in patients at stage IV trended to be less appreciable. Since the statistical significance of CSS cannot be seen between the young aged < 50 and age 50–59 group, age couldn't be identified as an independent prognostic factor in patients with stage IV SCLC. This discrepancy might present the increased lethality of SCLC at extensive stage. Several studies documented significantly worse survival and higher relapse rate of ED-SCLC than LD-SCLC [5, 32], demonstrating its extreme malignancy. In order to suppress the rapid development of distant metastases, it was commonly recognized that systematic chemotherapy was superior to local radiation in the treatment of ED-SCLC. In a phase 3 randomized controlled trial about ED-SCLC, additional thoracic radiotherapy after response to initial chemotherapy improved the survival rate of 2 years indeed, the overall survival wasn't prolonged [33]. Even though aggressive treatments have been provided due to their physical capacity, young patients didn't benefit significantly from the additional treatments. We further performed CSS analysis according to surgery condition among different age groups. Significant difference benefiting youth was only achieved in patients who underwent non-surgical treatment. For patients with resectable SCLC, outcomes after surgery were comparable to those with resectable NSCLC [34] and even better than SCLC patients who didn't receive surgery [35]. Our study might demonstrated that the benefit of surgery didn't exist in all ages. In the contrary, CSS significantly decreased with advanced ages in patients managed non-operatively. This result could present the survival characteristic of patient with unresectable SCLC in stage III and IV who predominated in patients with non-surgical treatment.

In addition, we performed CSS analysis according to radiation condition among different age groups. As we all known, the performance status of patients affects the choice of treatment including radiotherapy and chemotherapy. The young patients may be have better prognosis due to good performance status and receive more treatment. In fact, our results have showed most young patients aged < 50 received radiation, and most old patients aged ≥ 80 did not receive radiation. Therefore, in order to eliminate the effect of treatment on prognosis, we conducted subgroup analysis based therapy. Interestingly, we found that no matter the patients who received radiation or not, the young patients aged < 50 all had apparent CSS benefit except for age group 50–59. The present study has its intrinsic limitations. First, the SEER database didn't capture the detailed cancer therapies (regimen and dose of chemotherapy, target delineation and dose fractionation of radiotherapy et al.). Thus, our analysis wasn't able to adjust for these important variables. Second, performance status was also not available which was thought to correlate with advanced ages and result in increased mortality. Ignorance of this confounding factor would lead to inaccuracy of the results. But the prognostic value of age on SCLC still remains convincing given the considerably adequate number of patients.

In summary, patients with SCLC aged < 50 hold advantages in both OS and CSS compared to elder patients. The difference of CSS benefiting younger patients was most obvious at stage III and little appreciable at stage IV. The prognostic value of age is precise in patients without operations, but not in patients undergoing surgeries. In addition, no matter the patients who received radiation or not, the young patients aged < 50 tended to have a better prognosis.

## MATERIALS AND METHODS

### Patient selection

In total, 18 population-based cancer registries were included in the current SEER database and approximately 28% of the population in the United States was covered [36]. Appropriate patients were identified with the use of the SEER*Stat software (SEER*Stat 8.3.2). All patients between 2010 and 2013 with pathologically confirmed SCLC were included. The inclusive criteria included: confirmed age, active follow-up and only one primary tumor. Patients with incomplete staging, unknown age, unknown cause of death, unknown survival months and died within 30 days after surgery were all excluded.

### Ethics statement

This study was performed on the basis of public data from the SEER and conformed to the Helsinki Declaration. We were allowed to access the files of SEER program research data with the reference number of 11304-Nov 2015. Since no personal identifying information was involved the informed consent was not required. This study was approved by the ethics committee of the Shandong Cancer Hospital affiliated to Shandong University.

### Statistical analysis

For all the patients, the following variables were analyzed: Age, Race, Sex, AJCC stage, Grade,. Surgery and Radiation. It should be noted that Grade is actually pathological grade The primary endpoints of this study was CSS which were extracted from the SEER database. CSS is a net survival measure representing survival of a specified cause of death in the absence of other causes of death. Baseline characteristics of different groups were compared using χ^2^ tests. Survival curves were generated with the use of Kaplan-Meier estimates. The differences among the curves were analyzed through the Log Rank test. Univariate and multivariate Cox proportional hazards regression models were established to evaluate comparative risks of mortality and subgroup analysis. All statistical tests were two-sided and results were considered statistically significant when a test of a *P*< 0.05 achieved. The statistical software SPSS 18.0 (SPSS, IL, Chicago) was used for all data analysis.
